# A systematic review of venous thromboembolism mechanical prophylaxis devices during surgery

**DOI:** 10.1007/s00423-023-03142-6

**Published:** 2023-10-18

**Authors:** Brianna Herring, Darren Lowen, Prahlad Ho, Russell Hodgson

**Affiliations:** 1 Department of Surgery, University of Melbourne, Epping, Australia; 2 Department of Anaesthesia & Perioperative Medicine, Northern Health, Epping, VIC 3076 Australia; 3 Department of Critical Care, Melbourne Medical School, The University of Melbourne, Parkville, Australia; 4 Department of Haematology, Northern Health, Epping, VIC 3076 Australia; 5 Australian Centre for Blood Diseases, Monash University, Melbourne, Australia; 6 Department of Medicine, Northern Health, University of Melbourne, Heidelberg, Australia; 7 Department of Surgery, Northern Health, Epping, VIC 3076 Australia

**Keywords:** Venous thromboembolism, Deep vein thrombosis, Pulmonary embolism, Mechanical prophylaxis, Intermittent pneumatic compression, Graduated compression stockings

## Abstract

**Purpose:**

Hospitalisation and surgery are major risk factors for venous thromboembolism (VTE). Intermittent pneumatic compression (IPC) and graduated compression stockings (GCS) are common mechanical prophylaxis devices used to prevent VTE. This review compares the safety and efficacy of IPC and GCS used singularly and in combination for surgical patients.

**Methods:**

Ovid Medline and Pubmed were searched in a systematic review of the literature, and relevant articles were assessed against eligibility criteria for inclusion along PRISMA guidelines.

**Results:**

This review is a narrative description and critical analysis of available evidence. Fourteen articles were included in this review after meeting the criteria. Results of seven studies comparing the efficacy of IPC versus GCS had high heterogeneity but overall suggested IPC was superior to GCS. A further seven studies compared the combination of IPC and GCS versus GCS alone, the results of which suggest that combination mechanical prophylaxis may be superior to GCS alone in high-risk patients. No studies compared combination therapy to IPC alone. IPC appeared to have a superior safety profile, although it had a worse compliance rate and the quality of evidence was poor. The addition of pharmacological prophylaxis may make mechanical prophylaxis superfluous in the post-operative setting.

**Conclusion:**

IPC may be superior to GCS when used as a single prophylactic device. A combination of IPC and GCS may be more efficacious than GCS alone for high-risk patients. Further high-quality research is needed focusing on clinical relevance, safety and comparing combination mechanical prophylaxis to IPC alone, particularly in high-risk surgical settings when pharmacological prophylaxis is contraindicated.

## Introduction

Venous thromboembolism (VTE), encompassing deep vein thrombosis (DVT) and pulmonary embolism (PE), carries a significant disease burden [1]. Hospitalisation is one of the biggest risk factors with surgery posing an additional risk factor due to intra and post-operative immobility [1, 2]. Prevention methods are ranked as the top intervention hospitals can make to improve patient safety [3, 4].

Venous thromboembolism prophylaxis reduces rates of VTE by 55–70% [1]. Pharmacological methods of prophylaxis, such as low molecular weight heparin (LMWH), are highly effective. However, this needs to be weighed against the risk of bleeding and may be contraindicated at the time of the operation [2]. Mechanical methods may be equally effective, do not increase bleeding risk, and are likely of high importance at the time of operation [5, 6]. Devices such as intermittent pneumatic compression devices (IPC) and graduated compression stockings (GCS) reduce thrombus formation by preventing venous stasis. Intermittent pneumatic compression mimics the skeletal muscle pump, promoting pulsatile blood flow in the deep veins and increasing fibrinolysis [7]. Graduated compression stockings increase the velocity and volume of venous flow in the deep veins [8]. While these devices are considered safe, they have been associated with pressure-related side effects, including skin ulceration and pressure necrosis, and are contraindicated in some patients [2, 9]. Graduated compression stockings may also contribute to an increased fall risk [10].

International guidelines are varied, and recommendations are low grade and based on low-quality evidence [9, 11, 12]. Given the frequent use of mechanical prophylaxis and uncertainty in clinical guidelines, the aim of this review is to compare the safety and efficacy of IPC and GCS devices both singularly and in combination for surgical patients for the purpose of finding the ideal mechanical prophylaxis when pharmacological prophylaxis is not being used.

## Material and methods

OVID (Medline) and Pubmed were searched for full-text English articles prior to July 2021 using the search strategy in Table 1, and citation lists of relevant articles were also screened. Screening took place in the first week of September 2021 independently by the first author, with any articles deemed indeterminate also reviewed by the senior author. Inclusion criteria were primary data articles, surgical patient population, and at least 2 treatment groups containing one of the following: IPC, GCS or a combination of IPC and GCS. Primary outcomes had to include the incidence of VTE. The number of patients, type of surgery, methodology of the use of mechanical devices and complications were also noted. Given the heterogeneity of surgery and methodology, only a descriptive comparison was performed for this review. Randomised controlled trials were assessed for risk of bias using the Risk of Bias 2 (RoB 2) tool [13]. The review was designed to conform to the PRISMA guidelines and recommendations set out by the Study Center of the German Society of Surgery [14]. The review was not pre-registered on PROSPERO.

**Table 1:** Search strategy

Subject headings	Mechanical prophylaxis	VTE and safety outcomes	Surgical patient population
Key words: Each subject heading was searched with the listed key words and combined with Boolean operator OR. The resulting subject strings were combined with the Boolean operator AND	“mechanical prophylaxis”	thrombo*	surg*
	“intermittent pneumatic compression”	embolism	postoperative
	“sequential calf compression”	embolus	intraoperative
	“pneumatic stocking*”	safe*	preoperative
	“compression stocking*”	complication*	
	ted*		
	intermittent pneumatic compression devices (Pubmed MeSH term)	thromboembolism, venous (Pubmed MeSH term)	
	compression stocking (Pubmed MeSH term)		

## Results

**Fig. 1:**
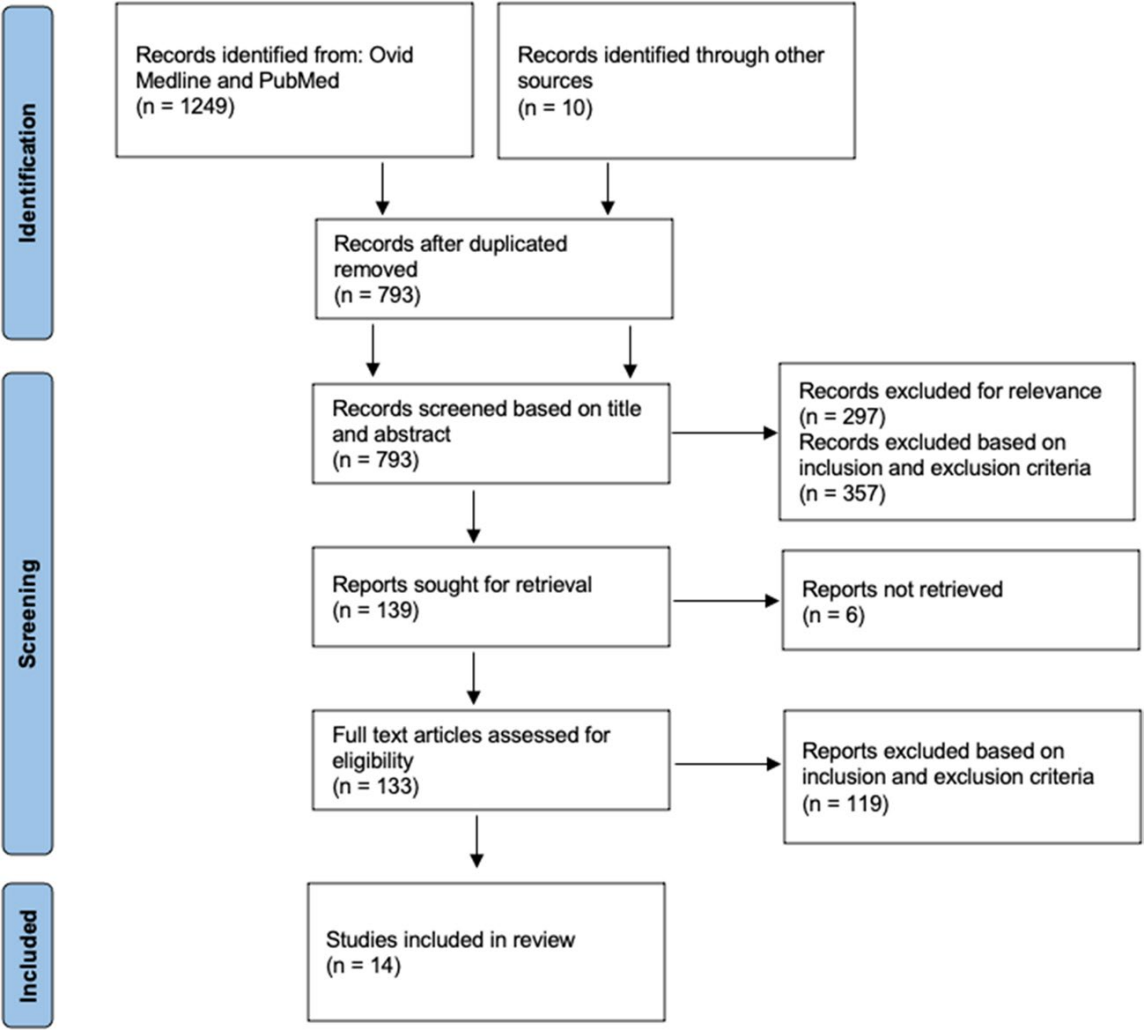
PRISMA flow diagram

The database search identified 1249 articles with an additional 10 identified from citation screening (Fig. 1). Titles and abstracts of 793 of these were screened, resulting in 139 articles for full-text screening. Five indeterminate articles were reviewed by the senior author, and all were excluded due to the fact they were primarily comparing chemical prophylaxis. Ultimately, 14 articles were included in this review, 12 randomised controlled trials and 2 retrospective cohort studies. The 12 randomised controlled trials were assessed for bias using the RoB 2 tool, as shown in Table 2, with only one study assessed as having an overall low risk of bias.

**Table 2:** Risk of bias (using the RoB2 tool [13])

Study	Randomisation process	Deviations from the intended interventions	Missing outcome data	Measurement of the outcome	Selection of the reported result	Overall rating
Pedegana et al. 1977 [15]	**-**	**!**	** + **	**-**	**!**	**-**
Van Arsdalen et al. 1983 [16]	** + **	**-**	** + **	**-**	**!**	**-**
Bucci et al. 1989 [17]	** + **	** + **	** + **	**-**	**!**	**-**
Ryan 2002 [19]	** + **	** + **	** + **	** + **	** + **	** + **
Silbersack et al. 2004 [20]	** + **	**!**	** + **	** + **	** + **	**!**
Chin et al. 2009 [21]	**!**	**!**	** + **	** + **	**!**	**!**
Turpie et al. 1989 [28]	** + **	** + **	** + **	**-**	** + **	**-**
Goldhaber et al. 1995 [27]	**!**	**!**	** + **	**!**	** + **	**!**
Gao et al. 2012 [23]	** + **	**-**	** + **	**-**	**-**	**-**
Prell et al. 2018 [25]	**!**	** + **	**-**	** + **	** + **	**!**
Sang et al. 2018 [26]	**!**	**!**	** + **	**!**	** + **	**!**
Lobastov et al. 2021 [24]	**-**	**-**	**!**	** + **	** + **	**-**

‘+’ Low risk; ‘!’ Some concerns; ‘-’ High risk

Of the fourteen included articles, seven studies compared IPC and GCS directly (Table 3) [15–21], and the remaining seven compared a combination of IPC and GCS versus GCS alone (Table 4) [22–28]. No studies were found that compared a combination of IPC and GCS against IPC alone. Six of the fourteen studies also included adverse events or compliance as secondary outcomes (Table 5).

**Table 3:** Key study characteristics and DVT and PE outcomes of studies comparing the efficacy of IPC and GCS devices

Study	Study type	Patient population	Intervention	DVT incidence	PE incidence
Pedegana et al. 1977 [15]	Randomised controlled trial	Total hip arthroscopy	IPC (*n* = 44)	0/44*	0/44*
			GCS (*n* = 56)	6/56 (11%)*	5/56 (9%)*
Van Arsdalen et al. 1983 [16]	Randomised controlled trial	Transurethral prostatectomy	IPC (*n* = 16)	2/16 (12.5%)	0/16 (0%)
			GCS (*n* = 21)	1/21 (4.8%)	1/21 (5%)
Bucci et al. 1989 [17]	Randomised controlled trial	Craniotomy	IPC (*n* = 38)	1/36 (5%)	-
			GCS (*n* = 37)	1/37 (5%)	-
Sarmiento et al. 1999 [18]	Retrospective cohort	Total hip arthroplasty	IPC (*n* = 718)	8/718 (1.25%)^a^	9/718 (1.25%)
			GCS (*n* = 774)	6/775 (0.78%)^a^	7/775 (0.9%)
Ryan 2002 [19]	Randomised controlled trial	Total hip arthroplasty	IPC (*n* = 50)	4/50 (8%)^b^*	0/50
			GCS (*n* = 50)	11/50 (22%)^b^*	0/50
Silbersack et al. 2004 [20]	Randomised controlled trial	Total hip replacement or total knee replacement	IPC + LMWH (*n* = 68)	0/68 (0%)*	-
			GCS + LMWH (*n* = 63)	18/63 (28.6%)*	-
Chin et al. 2009 [21]	Randomised controlled trial	Total knee arthroscopy	IPC (*n* = 110)	9/110 (8%)*^c^	0/110 (0%)
			GCS (*n* = 110)	14/110 (13%)^c^	1/110 (0.01%)
			No treatment (*n* = 110)	24/110 (22%)*	1/110 (1%)

^*^Statistically significant difference (*p* < 0.05)

^a^Symptomatic DVT only

^b^Proximal DVT only

^c^No statistical analysis performed

**Table 4:** Key study characteristics and DVT and PE outcomes of studies comparing the efficacy of combination mechanical prophylaxis

Study	Study type	Patient population	Intervention	DVT incidence	PE incidence
Turpie et al. 1989 [28]	Randomised controlled trial	Potential neurosurgical patients	GCS alone (*n* = 80)	7/80 (8.8%)*	-
			GCS + IPC (*n* = 80)	7/78 (9.0%)^+^	-
			No treatment (*n* = 81)	16/81 (19.8%)*^+^	-
Goldhaber et al. 1995 [27]	Randomised trial	Coronary artery bypass surgery	GCS + IPC (*n* = 172)	31/172 (19%)	-
			GCS alone (*n* = 172)	36/172 (22%)	-
Gao et al. 2012 [23]	Randomised trial	Gynaecological pelvic surgery, high-risk patients	IPC + GCS (*n* = 52)	5/104 (4.8%)^a^*	1/104 (1%)
			GCS (*n* = 56)	14/112 (12.5%)^a^*	1/112 (1%)
Chibbaro 2018^b^ [22]	Retrospective cohort	Neurosurgical patients	IPC + GCS + LMWH (*n* = 3818)	32/3818 (0.8%)	7/3818 (0.81%)
			GCS + LMWH (*n* = 3169)	73/3169 (2.3%)	28/3169 (0.9%)
Prell et al. 2018 [25]	Randomised trial	Craniotomy	GCS + IPC + LMWH (*n* = 41)	3/41 (7.3%)*	-
			GCS + LMWH (*n* = 53)	14/53 (26.4%)*	-
Sang et al. 2018 [26]	Randomised trial?	Gynaecological pelvic surgery	GCS (*n* = 159)	14/159 (8.8%)*	7/159 (4.4%)*^+^
			GCS + LMWH (*n* = 157)	6/157 (3.8%)	1/157 (0.64%)*
			GCS + IP (*n* = 153)	8/153 (5.2%)	3/153 (2.0%)
			GCS + IPC + LMWH (*n* = 156)	4/156 (2.6%)*	1/156 (0.65%)^+^
Lobastov et al. 2021 [24]	Randomised controlled trial	Major surgery, extremely high-risk patients	IPC + GCS + LMWH (*n* = 204)	1/204 (0.5%)*	0/204 (0%)
			GCS + LMWH (*n* = 203)	34/203 (16.7%)*	5/203 (2.5%)

^*,+^Statistically significant difference (*p* < 0.05)

^a^Incidence of DVT in legs

^b^No statistical analysis performed

**Table 5:** Secondary outcomes of studies comparing the efficacy of mechanical prophylaxis

Study	Intervention	Rate of adverse events	Rate of non-compliance	Comments
IPC vs. GCS				
Silbersack et al. 2004 [20]	IPC + LMWH (*n* = 68)	-	27%	The majority of IPC was not used correctly at the start of the study (cuffs not applied correctly, system not turned on)
	GCS + LMWH (*n* = 63)	-	-	
Chin et al. 2009 [21]	IPC (*n* = 110)	0	-	
	GCS (*n* = 110)	0	-	
	No treatment (*n* = 110)	0	-	
Combination of IPC + GCS vs. GCS alone				
Turpie et al. 1989 [28]	GCS alone (*n* = 80)	-	3%	2 patients did not wear according to the protocol
	GCS + IPC (*n* = 80)	-	13%	10 patients did not tolerate IPC, and 8 of these continued to wear GCS according to the protocol
	No treatment (*n* = 81)	-	-	
Goldhaber et al. 1995 [27]	GCS + IPC (*n* = 172)	-	36%*	Non-compliance is defined as > 3 h interruption to the protocol
	GCS alone (*n* = 172)	-	3%*	
Gao et al. 2012 [23]	IPC + GCS (*n* = 52)	0	-	
	GCS (*n* = 56)	0	-	
Sang et al. 2018 [26]	GCS (*n* = 159)	0	-	Adverse events related to mechanical devices only, bleeding complications not included
	GCS + LMWH (*n* = 157)	0	-	
	GCS + IPC (*n* = 153)	0	-	
	GCS + IPC + LMWH (*n* = 156)	0	-	
Lobastov et al. 2021 [24]	IPC + GCS + LMWH (*n* = 204)	12.3%	-	Adverse events defined as “leg skin injury”
	GCS + LMWH (*n* = 203)	7.4%	-	

^*^Statistically significant difference (*p* < 0.05)

### IPC vs. GCS

Of the seven studies comparing IPC and GCS directly, three randomised controlled trials demonstrated a significant difference in VTE rates (Table 3) [15, 19, 20]. Pedegana et al. reported 6/56 DVT and 5/56 PE in the GCS group, with none of either in the IPC group [15]. Similarly, Ryan et al. found the rates of proximal DVT to be significantly lower in the IPC group (8%) versus the GCS group (22%, *p* < 0.05) [19]. Silbersack et al. reported no incidence of DVT for those in the IPC group compared to the GCS group (28.6%, *p* < 0.0001) [20]. The remaining studies found no significant difference between IPC and GCS [16–18, 21].

### IPC + GCS vs. GCS alone

Seven compared a combination of IPC and GCS versus GCS alone (Table 4) [22–28]. Three randomised controlled trials found significantly lower rates of DVT with a combination mechanical prophylaxis [23–25]. Gao et al*.* reported that the rate of DVT in patients receiving IPC + GPC was 4.8% compared with GCS alone at 12.5% (*p* < 0.05) [23]. Prell et al. reported that the rate of DVT in patients receiving LMWH was significantly lower with the addition of combination mechanical prophylaxis (7.3%) versus GCS (26.4%, *p* < 0.05) [25]. Similarly, Lobastov et al. reported significantly lower rates of DVT with the addition of combination prophylaxis (0.5% versus 16.7%, *p* < 0.05) [24]. Chibbaro et al. reported lower rates of DVT and PE in patients receiving IPC and GCS (0.8% and 0.81%) compared to GCS alone (2.3% and 0.9%) [22]. The remaining studies did not demonstrate a significant difference in VTE events [26–28].

## Discussion

Recent studies have demonstrated the effectiveness of chemoprophylaxis alone for the prevention of DVT and PE [29]. However, there is emerging evidence that heparin or LMWH at the time of surgery increases bleeding complications in a range of operations [30–32]. With surgery being a critical time for thromboprophylaxis and mechanical devices showing no appreciable increase in bleeding risk, the identifiable gap in knowledge is the optimum mechanical thromboprophylaxis to be used during the operative period.

IPC and GCS are effective methods for reducing the risk of VTE in surgical patients without increasing the risk of bleeding [33–35]. These devices are generally considered safe; however, they have been associated with local tissue injury, nerve injury, compartment syndrome, and risk of falls [36]. Additionally, compliance is a considerable issue with mechanical prophylaxis which may affect their efficacy and risk of adverse events [37, 38]. Of note, the type of IPC and GCS devices differed between studies reviewed, and this may have an impact on efficacy, safety, and compliance [39, 40].

### IPC vs. GCS

Of the total of 7 studies identified, only three studies demonstrated a significant difference in VTE rates between the devices, all in favour of IPC (Table 3) [15, 19, 20]. Pedegana et al. found that the rate of both DVT and PE was significantly lower in the IPC group compared to GCS [15]. However, there were significant differences in patient characteristics between the treatment groups, with age and previous DVT being higher in the GCS group. Additionally, as with three other studies comparing IPC and GCS directly [15–18], this evidence is over 20 years old, and therefore, the applicability of these results to current practice is uncertain, given the changes in device design and manufacturing. A more recent trial by Silbersack et al. also found that IPC was associated with significantly lower rates of DVT compared to GCS [20]. However, IPC was commenced post-operatively and continued for 14 days, longer than other trials that varied between four and seven days post-operatively [15, 19, 21]. While a longer duration of device usage could have contributed to the superiority of ICP over GCS in this trial, there are major challenges in delivering IPC prophylaxis for 14 days, including physiotherapy and mobilisation, compliance and delivery post-discharge. However, there was no incidence of DVT in the 27% of patients who ceased IPC usage early due to increased mobility.

Sarmiento et al. found no significant difference in the rate of PE or DVT between patients receiving IPC or GCS [18]. The rate of symptomatic DVT for all groups was 1.2%, lower than trials by Predegana et al. and Silbersack et al. (6–14%), where all patients were investigated with ultrasound [15, 20]. These later studies, by investigating all patients, have detected both symptomatic and asymptomatic DVTs. The relevance (or lack thereof) of asymptomatic DVT is important, given that these studies found a significant difference in favour of IPC. Asymptomatic DVT is used as a surrogate outcome for symptomatic DVT to reduce study populations, and meta-analyses suggest a consistent relationship between relative changes in asymptomatic DVT and clinically relevant VTE [41–43]. However, there is some debate over the use of this surrogate outcome when weighing up risk and benefit, especially considering the difference in efficacy between agents is small [44–46].

Asymptomatic proximal but not distal DVT may be associated with an increase in all-cause mortality compared with no DVT [47–50], with the only evidence suggesting proximal DVT is significantly lower with IPC [19]. Thus, if a clinically meaningful difference in efficacy did exist in asymptomatic proximal DVTs, then a clinically meaningful difference between the devices may also exist.

A meta-analysis by Ho et al. pooled data from 9 studies and concluded that ICP is superior to GCS in reducing the risk of DVT (RR 0.61%) based on what they termed moderate quality evidence, although this is debated by Morris et al. [33, 51]. Undoubtedly, a high level of heterogeneity exists, particularly with the level of risk between patient cohorts, use and type of devices, and differences between symptomatic and asymptomatic measurements, thus leaving the superiority of IPC over GCS alone yet to be confirmed.

### Combination of IPC and GCS

Intermittent pneumatic compression and GCS are often used in combination, yet there are no recommendations regarding this practice. Unsurprisingly, a trial by Turpie et al. demonstrated a significantly lower rate of DVT with a combination of IPC and GCS compared to no prophylaxis [28]. The trend among seven further trials comparing combination mechanical prophylaxis to GCS alone was consistent, with all favouring combination prophylaxis [22–28]. However, statistical significance was reached in only three of these studies, and some of these studies were confounded by the treatment of patients with LMWH [23–25].

Lobostov et al., in a trial in extremely high-risk surgical patients, defined as having a Caprini score > 11, found the rate of DVT to be significantly lower with IPC and GCS compared to GCS alone [24]. Goa et al. found similar results, defining high risk upon the presence of risk factors such as history of VTE, hypercoagulability, heart disease, varicose veins or age greater than 60 [23]. However, in a trial by Goldhaber et al. where high-risk patients (those with a history of peripheral vascular disease, previous VTE or cardiac surgery) were excluded, there was no significant difference in DVT rates between combination mechanical prophylaxis and GCS alone [27].

Sang et al. risk-stratified patients within 4 treatment groups, but similarly found no statistical difference within any group other than a significant difference seen in the rates of DVT and PE in patients receiving combined mechanical prophylaxis and LMWH compared to GCS alone in the very high-risk patients (defined as having > 4 risk factors) [26]. Thus, the available evidence suggests that a combination of IPC and GCS may be superior to GCS alone for high-risk patients.

### Safety

Unlike pharmacological prophylaxis, mechanical prophylaxis devices do not increase the risk of major bleeding; however, they do carry a risk of local adverse events [36]. Skin ulceration is a particular issue regarding GCS use, and for this reason, GCS is contraindicated in patients with peripheral arterial disease or sensory impairment [2]. There are case reports in the literature of peroneal nerve injury, pressure necrosis and compartment syndrome associated with IPC [52–55].

Adverse events have only been investigated as secondary outcomes in a few studies comparing mechanical prophylaxis devices (Table 5) [21, 23, 24, 26]. Most report no adverse events [21, 23, 26]. However, in a trial by Lobastov et al. comparing combined IPC, GCS and LMWH with GCS and LMWH, the rates of skin injury in the legs were 12.3% and 7.4%, respectively, with no significant difference [24]. The overall numbers are inconsistent with previous large-scale trials of GCS and IPC which have demonstrated rates of adverse events of 5% and 1.3–2.9%, respectively [29, 56, 57].

A study in Australian hospitals found that 14% of hospital-acquired pressure injuries were associated with GCS and often occurred due to ill-fitting stockings, lack of staff awareness, and skin under-stockings not being assessed [58]. Therefore, while it is clear that mechanical thromboprophylaxis devices are associated with adverse outcomes, it is difficult to accurately measure this or compare devices, with observational studies with the best evidence available.

### Compliance

Compliance with mechanical prophylaxis devices is known to be a significant issue, particularly with IPC, where adherence has been reported between 40 and 89% [37, 38]. Compliance is influenced by both patient factors (discomfort and mobilisation) and health professional factors (knowledge and training regarding device usage) [37, 38].

No trial compared compliance with IPC and GCS directly. However, Turpie et al. found that in patients wearing both devices, where the rate of non-compliance was higher, the reason for discontinuation was intolerance of IPC devices, and 8/10 of these patients continued wearing GCS [28]. Goldhaber et al. also found that non-compliance was significantly higher in patients with the combination of GCS and IPC compared to GCS alone [27]. Interestingly, there remained no significant difference in DVT rates between the compliant and non-compliant groups. Increased mobility is a common reason for non-compliance, and given IPC devices mimic the skeletal muscle pump, non-compliance due to mobility may not impact DVT rates [37, 38].

### Mechanical and pharmacological prophylaxis

Although pharmacological prophylaxis was not the focus of this review, it is important to consider it, given many studies included LMWH in their treatment groups. While the American College of Chest Physicians (ACCP) guidelines generally prefer IPC over GCS, they recommend either device when used in combination with pharmacological prophylaxis [12]. However, while results from the recent Cochrane review have demonstrated that combined IPC and pharmacological prophylaxis is superior to either alone [35], trials have consistently been unable to demonstrate the superiority of LMWH and GCS over GCS alone [59–61]. This has led to a progression toward IPC rather than GCS for mechanical prophylaxis [62].

Sang et al. found that rates of DVT were significantly reduced in treatment groups with mechanical and LMWH compared to mechanical prophylaxis alone [26]. When analysed within risk groups, the difference remains significant for high-risk groups only, consistent with the American Society of Hematology (ASH) and ACCP guidelines [11, 12].

There have been high-powered studies demonstrating a lack of efficacy for mechanical thromboprophylaxis in non-surgical patients who are treated with prophylactic heparin or LMWH [29, 56]. This has now been confirmed in surgical patients with the GAPS study, which found no difference in thromboembolism in LMWH + GCS-treated post-surgical patients versus LMWH alone [61]. This may explain why studies have failed to find differences in thromboembolism rates when pharmacological prophylaxis is a constant. The importance of this cannot be understated as it could well mean that once surgical patients have commenced pharmacological prophylaxis, mechanical prophylaxis may not be required.

### Comparing current guidelines and limitations

There are a limited number of published guidelines that reference the different modes of mechanical thromboprophylaxis in surgical patients. The American Society of Hematology 2019 guidelines recommend IPC over GCS if mechanical prophylaxis is used, but make no mention of using both combined, and rate their recommendation as a conditional recommendation based on very low certainty in the evidence [11]. Similarly, the oft-used American College of Chest Physicians (ACCP) guidelines recommend IPC over GCS but are now slightly dated [12]. More localised guidelines, such as the NICE guidelines, simply suggest one or the other [9]. No commonly used international guidelines discuss using combined IPC and GCS, and therefore, no recommendations regarding this treatment exist. The available guidelines are in agreeance with the literature as reviewed in this study.

This study is limited by the use of English-only literature. The heterogeneity of the literature also hampered the ability to compare between studies, thus limiting the review to a narrative review only. Of the main papers reviewed, although most were randomised controlled trials, they tended to have questionable or high degrees of bias, with only one paper rated as having a low risk of bias. Of note, we initially performed this review for clinical reasons (hence lack of pre-registering), and our hospital has since changed policies to fit surgical patients with IPC only during operations, and therefore, we must acknowledge the potential for evidence selection bias.

## Conclusion

It is somewhat surprising, given the everyday practice of thromboembolism prophylaxis, that there is little evidence on which to guide practice. This is reflected in the variations in published clinical guidelines. Although some of the published literature is dated and heterogeneity exists, it appears that IPCs are superior to GCS in both preventing thromboembolism and in safety profile. However, the compliance and likely patient satisfaction with IPC if used post-operatively are worse. Combination therapy may be of advantage in high-risk patients, although with no comparison between combination therapy and IPC alone, it is difficult to make this judgement. With recent literature suggesting pharmacological prophylaxis alone may be appropriate for post-operative patients, further studies that assess mechanical prophylaxis, particularly comparing combination mechanical prophylaxis to IPC alone during the clinically relevant operative period and in those for whom pharmacological treatment is contraindicated, are also required.
